# Discovery and Characterization of Novel Vascular and Hematopoietic Genes Downstream of *Etsrp* in Zebrafish

**DOI:** 10.1371/journal.pone.0004994

**Published:** 2009-03-24

**Authors:** Gustavo A. Gomez, Matthew B. Veldman, Yan Zhao, Shawn Burgess, Shuo Lin

**Affiliations:** 1 Department of Molecular, Cell and Developmental Biology, University of California Los Angeles, Los Angeles, California, United States of America; 2 Genome Technology Branch, National Human Genome Research Institute, National Institutes of Health, Bethesda, Maryland, United States of America; Harvard Medical School, United States of America

## Abstract

The transcription factor Etsrp is required for vasculogenesis and primitive myelopoiesis in zebrafish. When ectopically expressed, *etsrp* is sufficient to induce the expression of many vascular and myeloid genes in zebrafish. The mammalian homolog of *etsrp*, *ER71/Etv2*, is also essential for vascular and hematopoietic development. To identify genes downstream of *etsrp*, gain-of-function experiments were performed for *etsrp* in zebrafish embryos followed by transcription profile analysis by microarray. Subsequent in vivo expression studies resulted in the identification of fourteen genes with blood and/or vascular expression, six of these being completely novel. Regulation of these genes by *etsrp* was confirmed by ectopic induction in *etsrp* overexpressing embryos and decreased expression in *etsrp* deficient embryos. Additional functional analysis of two newly discovered genes, *hapln1b* and *sh3gl3*, demonstrates their importance in embryonic vascular development. The results described here identify a group of genes downstream of *etsrp* likely to be critical for vascular and/or myeloid development.

## Introduction

The cardiovascular system of vertebrates provides a means to transport nutrients to and waste away from cells throughout the organism. As such, this system is critical for the survival of the organism. The cardiovascular system is made up of the vasculature and the blood that flows through it. It has been proposed that a common cell type, known as hemangioblasts, contribute to the development of vascular and blood cells [1], [2], [3], [4], [5]. This idea is supported by experimental evidence from several different organisms, notably the derivation of both blood and vascular endothelial cells from cultured mouse embryonic stem cells [6], [7], [8], and through in vivo lineage tracing studies in zebrafish and chick/quail chimeras [9], [10]. The zebrafish mutant line *cloche* provides genetic evidence since homozygous mutants lack both hematopoietic and vascular endothelial cells but organogenesis is otherwise normal [11]. As bi-potential precursor cells, hemangioblasts produce cells with more restricted potentials, angioblasts and hematopoietic progenitors [3]. Angioblasts subsequently produce the endothelial cells that line the vasculature during both vasculogenesis and angiogenesis, while hematopoietic progenitors differentiate into distinct blood lineages through hematopoiesis. Although some of the molecular factors that orchestrate these processes are known, many remain to be identified and studied.

Microarray studies were previously used to identify multiple novel hematopoietic and vascular genes misexpressed in the *cloche* mutant [12], [13], [14]. A particularly important gene identified in these studies was the transcription factor, Ets1-related protein (Etsrp), which is both necessary and sufficient to direct the development of the vascular and primitive myeloid lineages [13], [15], [16]. In zebrafish, hematopoietic/vascular development is separated into two distinct anatomical locations, the anterior lateral mesoderm (ALM) and posterior lateral mesoderm (PLM). The ALM gives rise to both primitive myeloid cells as well as the endothelial cells of the head vasculature. The PLM gives rise to primitive erythroid cells and the endothelial cells of the trunk and tail. *Etsrp* is expressed in both the ALM and PLM. Loss of *etsrp* function by morpholino antisense or genetic mutation, termed *y11*, results in loss of primitive myeloid cells and disrupted vasculogenesis and angiogenesis in the head and trunk. The defective vasculature in *etsrp* knockdown or mutant fish appears to be due to altered gene expression and cell behavior and not simply a loss of cells as the number of *fli:gfp* transgene positive cells is similar to control animals [15]. In contrast, the complete loss of *pu.1* staining in *etsrp* knockdown and mutants suggests that primitive myeloid cells are never specified [16], [17]. Interestingly, the primitive erythroid population in the PLM appears relatively normal when *etsrp* function is blocked. Thus, *etsrp* is critical for primitive myeloid and endothelial development from the ALM and endothelial development from the PLM but not the primitive erythroid cell population of the PLM.

A mammalian homolog of *etsrp, ER71/Etv2*, has recently been identified. *ER71/Etv2* knockout mice exhibit loss of vasculature and primitive erythrocytes suggesting it functions in the developing hemangioblast [18]. Additionally, overexpression of *ER71/Etv2* in zebrafish embryos causes the ectopic induction of the hemangioblast marker *scl/tal1* and the *flk1:gfp* transgene, identical to the results of *etsrp* overexpression. Therefore, both *etsrp* and *ER71/Etv2* play an evolutionarily conserved role in hematovascular development.

As members of the Ets transcription factor gene family, Etsrp and ER71/Etv2 have a conserved DNA binding domain and presumably act as transcriptional activators. Limited *in situ* hybridization analysis of *etsrp* morpholino gene knockdown or *y11* mutant embryos demonstrate that *etsrp* is necessary for the expression of *flk1*, *scl, fli1, pu.1* and a few other known genes specific to vascular and hematopoietic cells [15], [19]. Additionally, overexpression of *etsrp* in zebrafish embryos can ectopically induce vascular and myeloid gene expression [16]. We therefore decided to search for novel blood and vascular related genes downstream of *etsrp* by analyzing expression profiles of zebrafish embryos overexpressing *etsrp*. Here we report the identification of 14 genes with little or no prior investigations that are expressed in blood and/or vascular endothelial cells and demonstrate the function of two of these genes, *hapln1b* and *sh3gl3*, in vascular development using morpholino antisense in transgenic zebrafish embryos.

## Results

**Figure 1:**
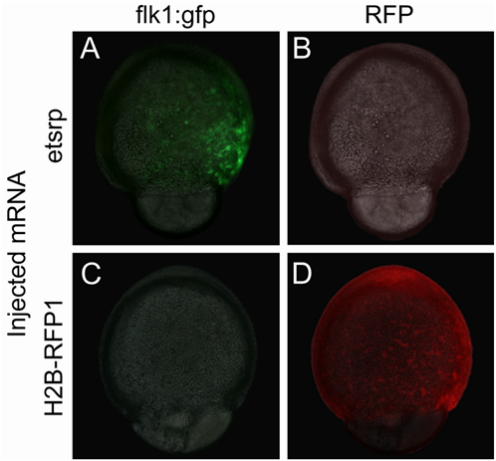
Early ectopic expression of transgenic *flk1:gfp* is induced by *etsrp* over-expression. (A) Injection of 75 pg of *etsrp* mRNA at one-cell stage results in the ectopic induction of *flk1:gfp* before the end of gastrulation. (B) No red fluorescence is observed in *etsrp* mRNA injected *flk1:gfp* transgenic embryos. (C) Injection of 500 pg of control *H2B-RFP1* mRNA does not result in the early induction of *flk1:gfp* similar to uninjected embryos (not shown). (D) Uniform H2B-RFP1 expression in a *H2B-RFP1* mRNA injected embryo. Panels A and C are composite images of light transmitted images merged with the green fluorescent channel, while panels B and D are composites of light transmitted images merged with the red fluorescent channel.

In order to identify novel blood or vascular related genes downstream of *etsrp*, the expression profiles of embryos ectopically expressing *etsrp* at late gastrulation stages, 80% epiboly to tail bud were compared to control embryos by microarray analysis. The *flk1:gfp* transgenic line was used to identify embryos successfully expressing *etsrp* since ectopic *etsrp* induces *flk:gfp* expression. Neither endogenous or transgenic *flk1:gfp* is normally expressed at 80% to tail-bud stage [20], [21], while early ectopic expression of *flk1:gfp* is evident in embryos injected with 75 pg of synthetic *etsrp* mRNA (Figure 1A). To ensure that the ectopic expression of *flk1:gfp* is induced by *etsrp* specifically, we injected up to 500 pg of synthetically transcribed mRNA encoding RFP tagged histone H2B, *H2B-RFP1*, as a control. Similar to uninjected controls, *flk1:gfp* expression is not induced in *H2B-RFP1* injected embryos (Figure 1C and 1D).

Prior to microarray analysis total RNA was extracted from pools of 70 embryos per treatment group and the change in expression of genes known to be induced by *etsrp* overexpression (OE) were assessed by quantitative RT-PCR. Both *fli1a* (11×) and *scl* (7.1×) were significantly induced by *etsrp* OE, while overexpression of *H2B-RFP1* resulted in no difference from uninjected controls (Figure S1). Gene expression analysis was then performed using a microarray chip containing 34,647 oligos representing approximately 20,000 genes. 686 genes were identified as being induced greater than two-fold by ectopic *etsrp* expression. Although some of the genes affected are not restricted to the circulatory system (Figure S2), a great number of previously identified hematopoietic and vascular genes were induced, validating the microarray strategy used in this study. Table 1 lists the 59 genes with the greatest fold induction as measured on the microarray.

**Table 1:** Top 59 genes induced by overexpression of *etsrp*.

GB accession	Unigene ID	Location	Gene name	Fold induction
BI980779	Dr.6315	chr14	Cdc42 guanine nucleotide exchange factor (GEF) 9	55.92
AI965222	Dr.45597	chr14	Cadherin 5 (cdh5)	54.96
DQ021472.1	Dr.47591	chr16	Ets1b/etsrp Ψ	47.01
AW233044	Dr.24158		Serglycin	39.62
NM_131626	Dr.81423		HAND2	34.52
AF045432	Dr.75812	chr22	Tal1/SCL	34.35
NM_152964	Dr.17548	chr6	Gastrulation brain homeo box 2 (Gbx2)	27.73
NM_131768	Dr.3499	chr3	Claudin I	26.36
AW420632	Dr.12726	chr21	Yes-relayed kinase	25.02
NM_131530	Dr.5729	chr11	Homeo box C6b	24.88
AI601685	Dr.5365	chr22	Dual specificity phosphatase 5 (dusp5)	24.52
BI673802	Dr.113768	chr11	Fgd5	23.26
AI477500	Dr.4847	chr11	Wnt7A	20.14
BI880801	Dr.83871	chr17	Rasgrp3	20.10
AW115759	Dr.23967	chr17	Complement receptor C1qR-like	19.35
AI477956	Dr.76040	chr8	Nexilin f actin binding	19.32
AW595297	Dr. 105109	chr8	UDP-GlcNAc:betaGal beta-1,3-N-acetylglucosaminyltransferase 3	18.21
Y14538	Dr.5730	chr19	Homeo box A9a	17.82
BI842764	Dr.14186	chr17	Si:ch211-214p16.1	17.50
AI793554	Dr.104443	chr16	Cellular retinoic acid binding protein 2a	15.98
BI882214	Dr.13039	chr15	Solute carrier family 43, member 2	14.62
BM181739	Dr.8453	chr2	PLA2-like otoconin	14.23
AI959344	Dr.28288	chr7	Matrix metalloproteinase precursor	13.34
BM036580	Dr.84866	chr16	Similar to hemicentin	12.93
AB055670	Dr.30464	chr23	Wnt1	12.77
Y14532	Dr.75789	chr12	Homeo box B6b	12.71
AI626374	Dr.2644		Dehydrogenase/reductase (SDR family) member 3	12.60
AI477237	Dr.77024	chr13	Similar to Olig3 protein	12.31
AW279740	Dr.22713	chr23	Zinc finger protein 385	12.18
BI982208	Dr.11052		Ornithine decarboxylase antizyme 2, like	11.42
BM103177	Dr.24950		Creatine kinase CKM3	11.27
AW076731	Dr.23392	chr25	integrin alpha, VLA protein type	10.63
AW233713	Dr.81043		Dusp2	10.56
BG892475	Dr.11064	chr2	amyloid precursor-like protein 2 (aplp2)	10.31
AA605884	Dr.38472		cDNA clone IMAGE:7137566	10.22
Y14527	Dr.5721	chr17	Homeo box A10b	10.15
NM_131359	Dr.75781		Bone morphogenetic protein 2a	9.90
NM_131419	Dr.272		NK2 transcription factor related 7	9.81
AI626636	Dr.45596		Adrenomedullin receptor (admr)	9.76
NM_131070	Dr.20373		Midkine-related growth factor	9.68
AW233616	Dr.121119		Calpain 8	9.52
AI522349	Dr.44232		Rhoub	9.46
NM_131000	Dr.20912		Activated leukocyte cell adhesion molecule	9.29
BG985561	Dr.8617	chr2	Hyaluronan and proteoglycan link protein 1b	9.21
NM_131767	Dr.26572		Claudin h (cldnh)	9.09
NM_131101	Dr.5572		Homeo box B5a	8.81
AF071249	Dr.28647	chr17	Homeo box A9b	8.47
BG727645	Dr.11977	chr16	Leucine rich repeat 33	8.00
BG985673	Dr.24989	chr10	Myosin light chain 2 like	7.80
Y13948	Dr.21032	chr9	Homeo box D3a	7.71
AI601355	Dr.77902		Similar to Fraser syndrome 1 isoform 1	7.61
NM_131419_1	Dr.272		NK2 transcription factor related 7	7.60
BM025541	Dr.3373	chr9	Collagen type XVIII, alpha 1	7.56
AF071568	Dr.20962	chr19	Homeo box B2a	7.43
AY028584	Dr.18294		Prostaglandin-endoperoxide synthase 1	7.38
AI601770	Dr.21376	chr23	Plexin precursor	7.27
AI721522	Dr.8674		Septin 9b	6.78
BG728550	Dr.81575	chr15	Cytochrome b5 reductase 4	6.58
AI964237	Dr.76050		ATP synthase, H+ transporting, mitochondrial F1 complex, alpha subunit 1	5.68

Note: Genebank accession number and unigene ID correspond to the probes on the array. The fold change column is the average ratio of eight measurements. Ψ The large induction ratio of *etsrp* reflects array probe hybridization to both the exogenously injected RNA as well as the induced expression of endogenous *etsrp*, (See Supplementary Figure 1).

From the genes affected by *etsrp* OE, we set up four criteria to select candidates for further studies: 1. They have not been well studied in hematopoiesis and/or vasculogenesis; 2. They are induced by ectopic *etsrp* in embryos as validated by whole mount *in situ* hybridization (WISH); 3. They are expressed in hematopoietic and/or vascular tissues; and 4. Expression is reduced by *etsrp* deficiency.

To identify genes with few or no prior studies for further analysis, the list of genes induced by *etsrp* OE was compared to the data sets for *cloche* mutants [13], [14]. We reasoned that genes identified in both *etsrp* OE and *cloche* mutant microarrays were highly likely to be involved in blood and/or vascular development. From this group of overlapping genes we screened for genes that had not been well studied using the PubMed database at the NCBI website. This resulted in the selection of 31 genes (Table 2), which were cloned and analyzed further.

**Table 2:** Genes screened for blood and/or vascular expression.

GB Accession	Unigene ID	Gene location	Gene name	Fold Induction	WISH for *etsrp* OE	down in *cloche*
BI980779	Dr.6315	chr14	Cdc42 guanine nucleotide exchange factor GEF 9 (arhgef9)	55.92	X	X
AW171479	Dr.2433	chr12	Carboxylesterase 2-like(cbe2)	3.32	X	X
**AF109780**	**Dr.81287**		**Kreisler maf-related leucine zipper homolog 2 (krml2)**	**4.82**	**X**	**X**
AF071255	Dr.117289	chr12	Hoxb8b	3.57	X	X
**BI880801**	**Dr.83871**	**chr17**	**RAS guanyl releasing protein 3 (rasrgrp3)**	**20.51**	**X**	
**BG727645**	**Dr.11977**	**chr16**	**Leucine rich repeat containing 33 (lrrp33)**	**8.00**	**X**	**X**
AW279740	Dr.22713	chr23	Zinc finger protein 385 (znf385)	12.18	X	
BI673729	Dr.14541	chr6	Similar to leucine rich repeat containing 51 (lrrp51)*	6.08	X	
AI544475	Dr.33183	chr13	SPARC related modular calcium binding 2 (smoc2)	6.68	X	
BI867572	Dr.9564	chr21	Similar to testis-expressed sequence 2(tex2)	4.91	X	
AW421939	Dr.5562	chr20	Apolipoprotein B (apolipo b)	3.07	X	
BI430050	Dr.418		Protocadherin2 gamma20 (pcdhg)	3.50		
BI672391	Dr.14449		weakly similar to XP_422395.1 (novel 2)	5.61	X	
BC093143	Dr.20125	chr1	hypothetical LOC550501	6.80		
**BI864031**	**Dr.13409**	**chr12**	**Anthrax toxin receptor 1/similar to tumor endothelial marker 8 (antxr1/tem8)**	**7.29**	**X**	
BM095233	Dr.17582	chr23	C1orf192 homolog	4.47		
**BI671403**	**Dr.120608**	**chr24**	**PTPL1-associated RhoGAP 1 (arhgap29)**	**5.65**	**X**	
**BI673802**	**Dr.113768**	**chr11**	**Fyve rhogef and ph domain containing zinc finger fyve domain containing protein 23 (Fgd5)**	**23.26**	**X**	**X**
AY028584	Dr.18294		Prostaglandin-endoperoxide synthase 1 (ptgs1)	7.38		
**BG985561**	**Dr.8617**	**chr2**	**Hyaluronan and proteoglycan link protein 1 precursor (hapln1B)**	**9.21**	**X**	
BI842967	Dr.76442	chr8	hypothetical LOC555375	3.68		
BI880784	Dr.12941		Similar to testican 3	3.81		
**AW420632**	**Dr.12726**	**chr21**	**Yes-relayed kinase (yrk)**	**25.02**	**X**	**X**
**BM036580**	**Dr.84866**	**chr16**	**Similar to hemicentin**	**12.93**	**X**	
**BI983776**	**Dr.12218**	**chr25**	SH3-domain GRB2-like 3 (sh3gl3) *	**2.76**	**X**	
**AI884043**		**chr24**	Similar to immune costimulatory protein *	**3.34**	**X**	
X60095	Dr.510	chr23	Hoxc3a	3.37		
**BI428793**			**Lim domain binding 2 (ldb2)**	**4.21**	**X**	
**AI721944**	**Dr.21605**	**chr3**	**Est- AI721944**	**2.92**	**X**	
**AW019729**		**chr8**	**Est- AW019729**	**2.68**	**X**	
AA542593	Dr.75525	chr23	C20.orf.112	5.09		

Note: Genebank accession number and unigene ID correspond to the probes on the array. The fold change column is the average ratio of eight measurements. (*) ectopic induction was observed for lrrp51 but no expression was detected between 24–30 hpf. Genes in bold indicate those that are expressed in blood or vascular endothelial cells at 24–30 hpf.

**Figure 2:**
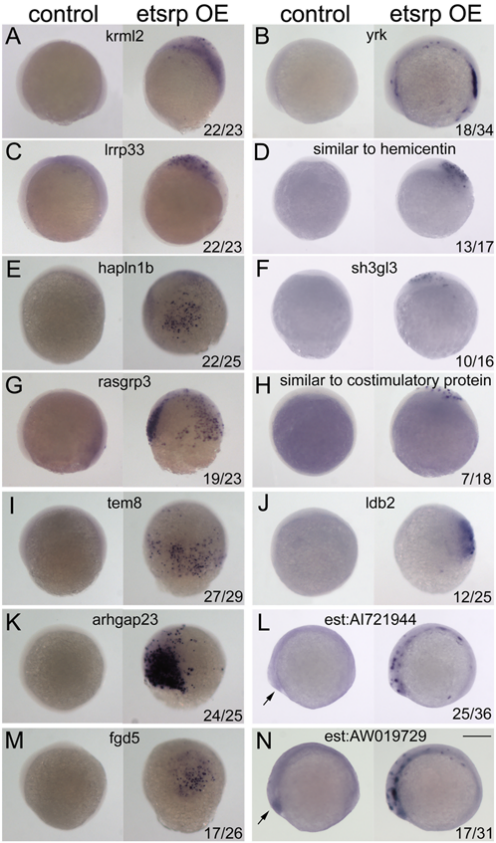
Ectopic induction of microarray identified genes by *etsrp* overexpression. Ectopic gene expression was examined at 80% epiboly to tailbud stages in *flk1:gfp* transgenic embryos injected with 30 pg *etsrp*-mcherry DNA at the one cell stage. Embryos exhibiting both red and green fluorescence were selected for analysis. Representative control embryos are on the left and *etsrp* overexpressing (OE) embryos are on the right side of each panel. Represented genes: (A) *krml2*; (B) *yrk*; (C) *lrrp33*; (D) *similar to hemicentin*; (E) *hapln1b*; (F) *sh3gl3*; (G) *rasgrp3*; (H) *similar to costimulatory protein*; (I) *tem8*; (J) *ldb2*; (K) *arhgap23*; (L) *est:AI721944*; (M) *fgd5*; and (N) *est:AW019729*. Note that there is a low level of endogenous expression in the control embryos for the two EST's, L and N, at the polster (arrows). Ratios in bottom right hand corner in panels represent the number of embryos with ectopic induction of total embryos processed and scored in the injected groups; control embryos never displayed ectopic induction. All embryos are in lateral view, and those at tail bud stage are oriented with anterior to the left. Scale bar: 250 µm.

To validate the results obtained with the *etsrp* OE microarray, WISH was performed on embryos at the same developmental stage as was done for the microarray, 80% epiboly to tailbud. To ensure that the OE samples examined were indeed ectopically expressing *etsrp*, embryos were injected with plasmid DNA encoding an Etsrp-mcherry fusion protein. This fusion protein was functionally active since it efficiently induced ectopic expression of *flk:gfp* when injected into transgenic embryos (data not shown). Embryos expressing Etsrp-mcherry and *flk:gfp* were selected to test for ectopic induction for 23 of the 31 genes identified in Table 2. 21 of 23 genes were clearly induced ectopically by *etsrp* OE. Figure 2 shows the ectopic induction of the 14 genes identified below to be relevant to blood and/or vascular expression. There was ubiquitous endogenous expression in 2/23 genes, *arhgef9* and *znf385*, prohibiting any qualitative discernment of their changes in expression by *etsrp* OE (data not shown). Overall, this demonstrates that the microarray data is low in false positives and reliable.

**Figure 3:**
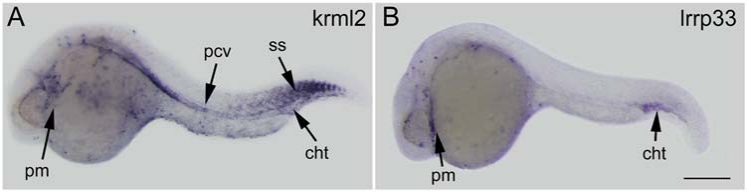
Expression of genes identified in primitive myeloid blood cells. Gene expression was examined at 24–28 hours post fertilization by whole mount in situ hybridization in wildtype embryos. (A) *krml2* is expressed in primitive myeloid cells (dispersed cells labeled throughout yolk and head) in the posterior cardinal vein, caudal hematopoietic tail region, and somites. (B) *Lrrp33* is expressed in primitive myeloid cells and in the caudal hematopoietic tissue Abbreviations: pm, primitive myeloid; pcv, posterior cardinal vein; cht, caudal hematopoietic tail region; ss, somites. Scale bar: 250 µm.

**Figure 4:**
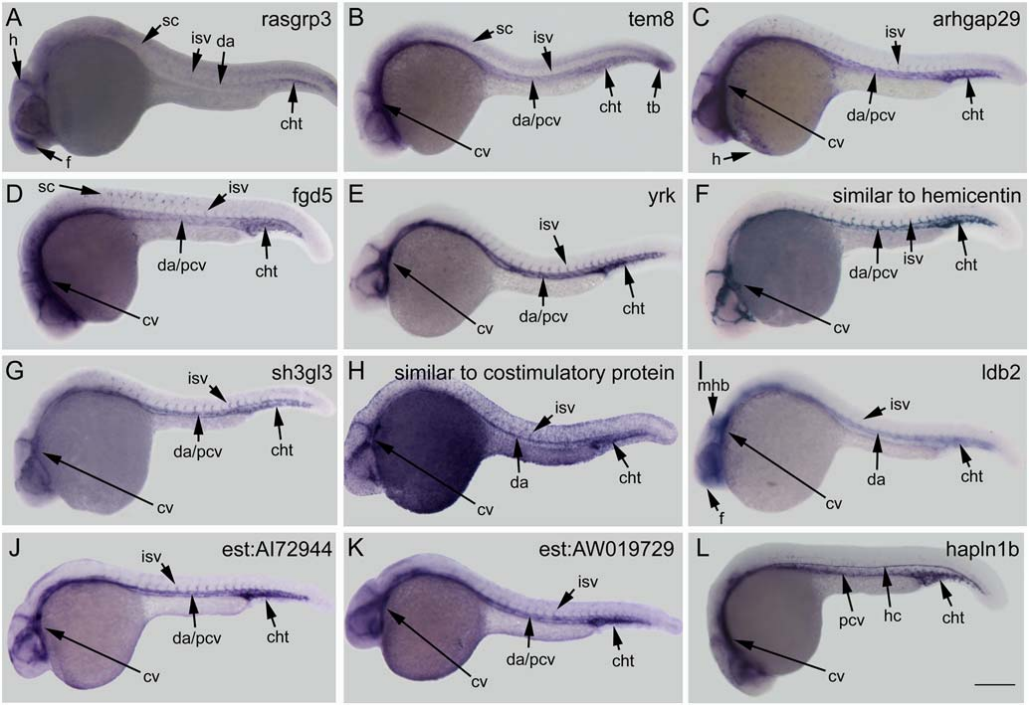
Expression of genes identified in vascular endothelial cells. Gene expression was examined at 24–28 hours post fertilization by whole mount *in situ* hybridization in wildtype embryos. (A) *rasgrp3* is expressed weakly in the forebrain, hindbrain, spinal cord, intersomitic vessels, dorsal aorta, and caudal hematopoietic tail region. (B) *tem8* is expressed highly in the cranial vasculature, the dorsal aorta and posterior cardinal vein, tailbud and weakly in the intersomitic vessels and spinal cord. (C) *arhgap29* is expressed in the cranial vessels, dorsal aorta, posterior cardinal vein, intersomitic vessels, caudal hematopoetic tail region, and hatching gland. (D) *fgd5* is expressed in the cranial vasculature, dorsal aorta, posterior cardinal vein, intersomitic vessels, and caudal hematopoetic tail region. (E, F, and G) *yrk*, *similar to hemicentin*, and *sh3gl3* are all expressed in the cranial vasculature, dorsal aorta, posterior cardinal vein, intersegmental vessels, and caudal vascular hematopoietic tail region. (H) *similar to costimulatory protein* is expressed in the cranial vasculature, dorsal aorta, intersomitic vessels, and caudal hematopoietic tail region; additional expression is present in the ectoderm layer throughout the embryo. (I) *ldb2* is expressed in the forebrain, midbrain-hindbrain boundary, dorsal aorta, intersomitic vessels, and caudal hematopoietic tail region. (J and K) Both *est:AI721944* and *est:AW019729* are expressed in the cranial vasculature, dorsal aorta, posterior cardinal vein, intersomitic vessels, and caudal hematopoietic tail region. (L) *hapln1b* is expressed in the cranial vasculature, posterior cardinal vein, caudal hematopoietic tail region, and hypochord. Abbreviations: f, forebrain; h, hindbrain; sc, spinal cord; cv, cranial vasculature; da, dorsal aorta; pcv, posterior cardinal vein; isv, intersomitic vessels; cht, caudal hematopoietic tail region; tb, tail bud; h, hatching gland; and hc, hypochord. Scale bar: 250 µm.

To screen the 31 selected genes for blood and/or vascular expression, their expression patterns were examined in wild type embryos by WISH at 24–30 hours post fertilization (hpf). Of the genes examined, two are expressed in blood lineages (Figure 3), and twelve in vasculature (Figure 4), while sixteen other genes demonstrate tissue specificity but are irrelevant to blood or vessels (Figure S2).

**Figure 5:**
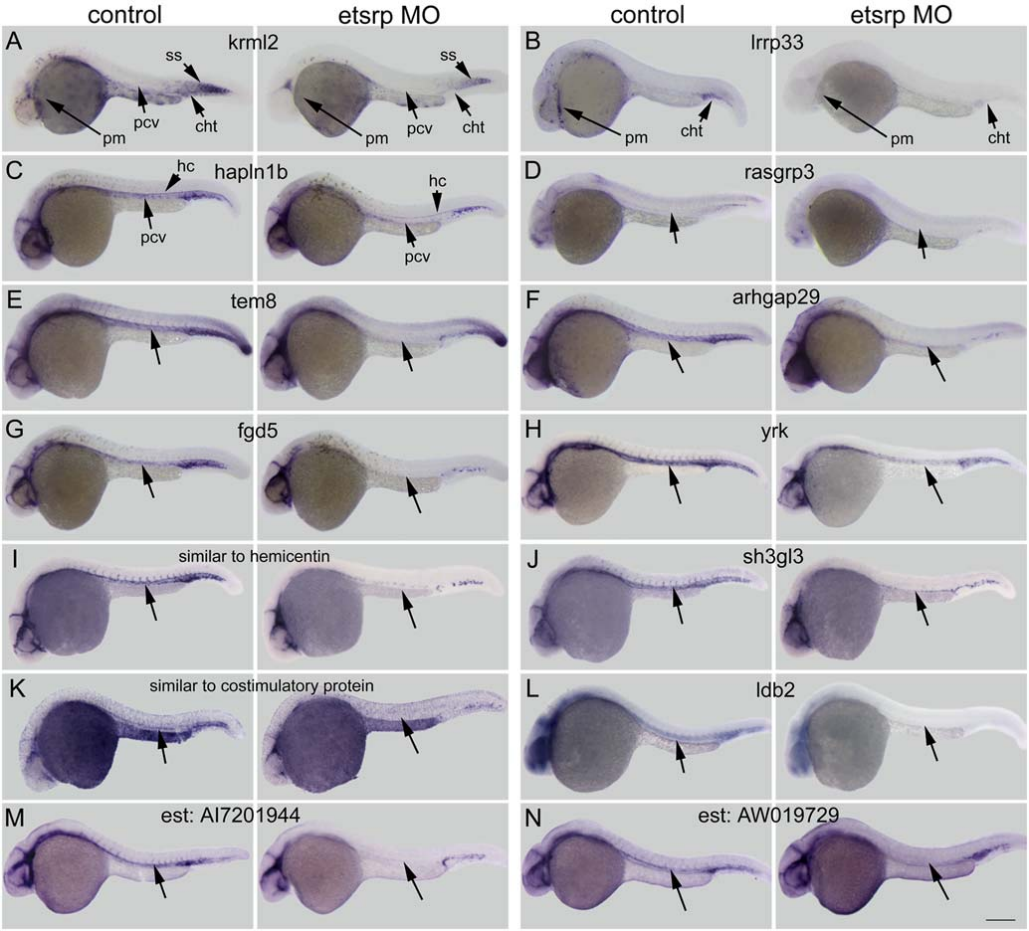
Morpholino knockdown of *etsrp* reduces expression of genes identified by *etsrp* overexpression microarray analysis. Embryos were injected with a combination of two *etsrp* targeting morpholinos (4 ng each) and gene expression was examined by in situ hybridization at 24–26 hours post fertilization. Representative control embryos are on the left of each panel and *etsrp* morpholino (MO) injected embryos are on the right. (A) *krml2* is reduced in primitive myeloid cells, posterior cardinal vein, and caudal hematopoietic tail region by *etsrp* knockdown. (B) *lrrp33* expression is absent in myeloid cells and reduced in the caudal hematopoietic tail region. (C) *hapln1b* expression is lost in the posterior cardinal vein when *etsrp* is knocked down. (D–N) *Etsrp* knockdown causes a significant decrease of gene expression, most pronounced in the trunk (unlabeled arrows). (D) *rasgfp3*; (E) *tem8*; (F) *arhgap29*; (G) *fgd5*; (H) *yrk*; (I) *similar to hemicentin*; (J) *sh3gl3*; (K) *similar to costimulatory protein*; (L) *ldb2*; (M) *est:AI7201944*; and (N) *est:AW019729*. Note that non-hematovascular tissues such as somites in (A) and hypochord in (C) are not affected by *etsrp* knockdown. Abbreviations: pm, primitive myeloid; pcv, posterior cardinal vein; cht, caudal hematopoietic tail region; ss, somites; and hc, hypochord. Scale bar: 250 µm.

The dependence of the new blood and vascular specific genes on *etsrp* was then examined by comparing their expression in *etsrp* morphants to uninjected control embryos (Figure 5). In *etsrp* morphants, the expression of *krml2* (Figure 5A) and *lrrp33* (Figure 5B) was abolished in myeloid cells, while the remaining 12 vascular genes were also significantly reduced in expression, most noticeably in the trunk region containing the dorsal aorta, posterior cardinal vein, and intersomitic vessels (Figure 5, C–N). Note that gene expression in the cranial vasculature was less affected in *etsrp* morphants but still reduced. Gene expression in tissues outside of blood and vessels such as somites for *krml2* (Figure 5A), neurons for *rasgrp3* (Figure 5D) and *ldb2* (Figure 5L), and tailbud for *tem8* (Figure 5E) were not affected by *etsrp* knockdown. This demonstrates that *etsrp* is not only sufficient but also required for normal expression in primitive myeloid and/or vascular cells in the 14 genes identified here.

### Genes downstream of *etsrp* with endogenous expression in blood

#### Krml2

Krml2 is a member of the Maf family of basic leucine zipper transcription factors that have high evolutionary conservation with orthologs existing in chicken, *Xenopus*, mice, and humans [22], [23]. The expression of *krml2* in zebrafish was previously found in somites, reticulospinal oculomotor neurons, and lens [24]. In this study we have also detected its expression in primitive myeloid cells and the vasculature (Figure 3A). The mouse and avian orthologs of *krml2* have also been detected in macrophages [25], and it has been demonstrated to regulate hematopoiesis by repressing erythropoiesis in favor of the myeloid fate through an interaction with Ets1 [26].

#### LRRP33

Leucine rich repeat containing protein 33 is a member of the leucine-rich repeat protein family. Lrrp33 is a single pass transmembrane protein that contains 21 leucine-rich repeats, and has orthologs in mice, rats, and humans, which remain to be characterized. The leucine-rich repeat family of proteins consists of over 60,000 members with diverse biological activities, but a common feature shared by proteins with LRR motifs is their propensity to associate with other proteins through LRRs [27]. Prominent members of this family with characterized roles in inflammatory and innate immunity are the toll-like receptors, which are comprised of ten members in humans, with homologs for all encoded in the zebrafish genome [28], [29]. *Lrrp33* is excluded from this subfamily because it lacks the cytosolic toll-interleukin (TIR) receptor domain through which TLRs transduce intracellular signals [30], [31]. In this study we have detected *lrrp33* expression in primitive myeloid cells and the caudal hematopoietic tail region (Figure 3B).

### Genes downstream of *etsrp* with endogenous expression in vasculature

#### RAS guanyl releasing protein 3 (RASgrp3)

RASgrp3 is a member of the RAS family of GTPases which link cell surface receptor activation and RAS activation by switching its state of activity between inactive GDP and active GTP bound states [32]. With orthologs in mice and humans, zebrafish *RASgrp3* encodes a protein with 708 residues containing an N-terminal and catalytic RhoGEF domains, followed by a pair of calcium binding EF-hand domains and a zinc dependent DAG/phorbol-ester binding domain. *RASgrp3* is expressed at low levels in both axial and cranial vasculature, and the caudal vascular plexus region, as well as the neural tube and hindbrain (Figure 4A). A role for *RASgrp3* in endothelial cells was initially identified in an embryonic stem cell gene trap assay [33]. In endothelial cells *RASgrp3* is up-regulated by VEGF signaling, and functions as a phorbol ester receptor in both B-cells and endothelial cells, where it has been ascribed with forming a disorganized angiogenic vasculature in disease states [33], [34]. There are currently four *RASgrp* genes encoded in mammals, *RASgrp-1*, *-2*, *-3*, and -*4*, while zebrafish only have three, *RASgrp-1*, -*2*, and -*3*. The mouse ortholog is expressed in the vasculature during developmental stages, but the knockout is viable [33].

#### Similar to tumor endothelial marker 8 (tem8)/Anthrax Receptor 1 (antxr1)

*Tem8* was initially identified as a gene up-regulated in tumors, and is one of two anthrax toxin receptors, the other being *capillary morphogenesis gene 2 (CMG2)/ANTXR2* which together recognize and internalize anthrax toxins [35], [36], [37]. Zebrafish *tem8* encodes a 552 amino acid transmembrane protein with a von Willebrand factor type A domain, an extracellular Ig anthrax toxin receptor domain and the cytoplasmic c-terminal anthrax receptor domain. It is expressed at low levels in neurons with higher expression levels in the tailbud as well as the axial and cranial vascular endothelial cells (Figure 4B). Two *cmg2* orthologs have been identified in zebrafish, *cmg2a* and *cmg2b*, but their expression remains to be examined [38]. *Tem8* regulates cell adhesion and migration by coupling extracellular stimuli to changes in the actin cytoskeleton [39], [40]. Regulation of cell adhesion and migration by *tem8* plays a role in angiogenesis and is relevant to tumor angiogenesis, anthrax toxin responses, and potentially circulatory system development.

#### RhoGTPase-activating protein 29 (arhgap29)

Arhgap29 was identified in a screen for proteins that interact with the protein-tyrosine phosphatase PTPL1 (which is expressed in many tissues), and has the alternative name PTPL1-Associated RHOGAP1 (PARG1) [41]. Zebrafish arhgap29 is a guanine activating protein of RhoGTPases that is 1365 amino acids long with a protein kinase C domain and a RhoGAP domain which are conserved in orthologs in multiple species including humans. The expression of *ARHGAP29* has been detected in the hearts of mouse embryos among other tissues [42], and in zebrafish it is expressed in the cranial vasculature, dorsal aorta, cardinal vein, intersomitic vessels, caudal vascular plexus, and hatching gland at 24 hpf (Figure 4C).

#### FYVE, rhoGEF and PH domain containing protein 5 (fgd5)

Fgd5 is a member of the Dbl homology (DH) protein family that functions as guanine nucleotide exchange factors for the RhoGTPases Rho, Rac and Cdc42 [43]. There are six paralogs of this gene in mammals. The family's name is derived from the founding member, *fgd1*, which causes faciogenital dysplasia in humans when mutated [44]. Fgd5 has been widely conserved through evolution and, in zebrafish, Fgd5 is 1603 amino acids long with the common domains of all DH family members, which includes a DH domain followed by two plextrin homology PH domains that flank a FYVE containing zinc finger domain. *Fgd5* is expressed in the endocardium of mice during development [45], while in zebrafish it is expressed throughout the vascular system, including the heart (Figure 4D).

#### Yes-related kinase (yrk)

*Yes-related kinase (yrk)* is a homolog of the *yes* oncogene, and is a member of the *src* non-receptor tyrosine kinase family. It is distinct from other *src* members including *fyn* and *yes*, and is expressed in the cerebellum, spleen, lung, and skin in adult chicken tissues [46]. The protein is composed of 528 amino acids with single SH2, SH3 and tyrosine kinase domains. Unlike its ortholog in chicken, the zebrafish *yrk* is expressed exclusively in vascular endothelial cells where its function remains to be investigated (Figure 4E).

#### Similar to hemicentin

Hemicentin is an extracellular matrix ECM protein of the immunoglobulin superfamily identified originally in *c.elegans* and found to facilitate tissue organization and cell migration [47]. The human ortholog has been associated with age related macular degeneration [48] and in mice its expression has been observed at the pericellular ECM of various epithelial cells including embryonic trophectoderm, skin, tongue, in addition to the ECM of some blood vessels [49]. Distinct from hemicentin, zebrafish similar to hemicentin differs in size, structure and possibly function. While hemicentin contains a von willberand A domain, 48 tandem Ig domains, multiple tandem epidermal growth factor repeats (EGFs), and a single fibulin-like C-terminal domain, zebrafish similar to hemicentin is 264 amino acids long, has a single Ig domain and a transmembrane domain. Its specific expression in the vascular system suggests a potential role for this gene in endothelial cells (Figure 4F).

#### SH3 domain Growth factor Receptor Bound protein 2-like 3 (sh3gl3)

*Sh3gl3* encodes for a protein that is 386 amino acids long which contains a Bin-Amphiphysin-Rvs (BAR) domain implicated in synaptic vesicle internalization, actin regulation, differentiation, and cell survival, and a src homology 3 (SH3) domain [50]. The mammalian SH3gl3, also referred to as endophilin A3 and EEN-B2, is a member of a small family of SH3 containing proteins known as the SH3GL family that includes three members, EEN, EEN-B1 and EEN-B2 [51]. In adult mice, *EEN-B2* is expressed in the brain, testis and spinal cord, while its embryonic expression is ubiquitous and there appears to be a marked transient expression in the vascular system [52]. In zebrafish, *sh3gl3* expression is vascular specific at the stage examined here (Figure 4G). Besides *sh3gl3*, paralogs in zebrafish include *sh3gl1a*, *sh3gl1b*, *sh3gl2, sh3gl2b, and zgc:158742*.

#### Similar to immune costimulatory protein

*Similar to immune costimulatory protein* is a novel gene that encodes a 372 amino acid long transmembrane protein with two tandem Ig-like domains at the N-terminus followed by a transmembrane domain. It is expressed in the cranial vasculature, the caudal vascular plexus, and is restricted to the artery in the axial vessels. It is also expressed in a spotted pattern throughout the ectoderm (Figure 4H).

#### LIM domain binding protein 2 (ldb2)

LIM domain binding protein 2 (ldb2) is a LIM homeodomain co-activator that is one of four *ldb* genes found in zebrafish. *Ldb2* has been reported to be induced in human vascular endothelial cells following stimulation with VEGF [53]. The expression of *ldb2* in zebrafish has been described in the central nervous system and vasculature [54], which we confirmed in Figure 4I. We have also found that *ldb2* is silenced in the axial vasculature of *etsrp* morphants (Figure 5L).

#### Novel Expressed Sequence Tags (ESTs)

The overexpression of *etsrp* resulted in the identification of two ESTs that could not be linked to any annotated genes but which are clearly induced ectopically (Figure 2L and 2N), demonstrate vascular specific expression, including cranial, axial, caudal and intersomitic vessels (Figure 4J and 4K), and whose expression is absent in the axial and intersomitic vessels in *etsrp* morphants (Figure 5M and 5N). Their accession numbers are: AI721944 and AW019729.

#### Hapln1b

Hyaluronan and proteoglycan link protein 1b is a member of the vertebrate hyaluronan and proteoglycan link protein family which consists of four members in mice and humans, *HAPLN-1*,*-2*,*-3*,-*4* [55]. Hapln1, also known as cartilage link protein, is a secreted extracellular matrix protein that stabilizes aggrecan-hyaluronan complexes in cartilage and other tissues, with the knockout in mice resulting in dwarfism and craniofacial abnormalities [56]. Zebrafish also contain four *hapln* genes, *hapln-1a*, -*1b*, -*2*, and *-3*. During early stages of zebrafish development, *hapln1b* is expressed in the hypochord, cranial vasculature, posterior cardinal vein, and caudal hematopoietic tail region (Figure 4L and [57]).

### Functional Analysis of *hapln1b* and *sh3gl3*

**Figure 6:**
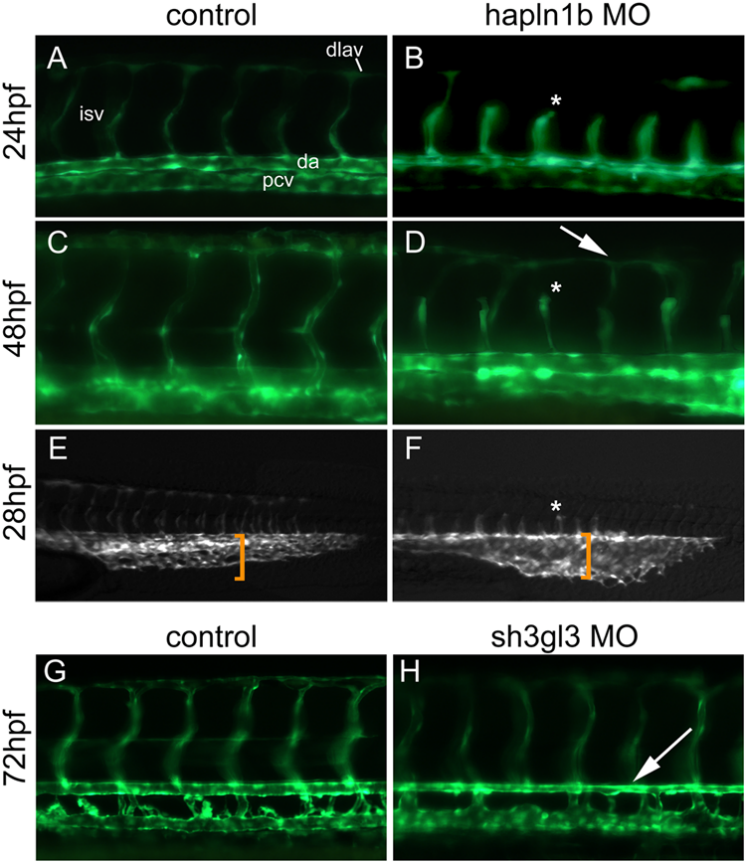
*Hapln1b* and *sh3gl3* are mediators of vascular development. (A–F) *Flk1:gfp* transgenic embryos injected with 2 ng of a translation blocking morpholino (MO) targeting *hapln1b* arrests angiogenic sprouting of the intersomitic vessels (asterisks in panels B, D, F; compare to their counterparts in A, C, and E respectively), resulting in delayed and improper dorsal longitudinal anastomotic vessel formation (arrow in D). Furthermore, the caudal vascular plexus is dilated relative to wild-type controls at 28 hours post fertilization (hpf) (compare the identical sized bracket in panels E and F). (G and H) Injection of 3 ng of translation blocking MO targeting *sh3gl3* results in a thinner dorsal aorta (arrow in H) relative to wild type controls (G) at 72 hpf. Abbreviations: dlav, dorsal longitudinal anastomotic vessels; isv, intersomitic vessels; da, dorsal aorta; and pcv, posterior cardinal vein.

To determine if the genes highlighted here might play a functional role in vascular development we used antisense morpholino (MO) oligos to knockdown *hapln1b* and *sh3gl3* in *flk:gfp* transgenic fish. *Hapln1b* knockdown using 2 ng of morpholino results in the arrested angiogenic sprouting of many intersomitic vessels (asterisks in Figure 6B, 6D and 6F), preventing them from forming stereotypical dorsolateral anastamosing vessels (dlavs) by 48 hpf (arrow in Figure 6D). Furthermore, the vascular remodeling that occurs at the caudal vascular plexus around 28–30 hpf is severely defective in *hapln1b* morphants (compare bracketed region in Figure 6E and 6F). These defects were observed in 87 out of 92 (92.6%) embryos injected with *hapln1b*-MO while standard control MO (5 ng) injected embryos never exhibited this phenotype (0 out of 70 embryos). *Sh3gl3* morphants (3 ng) had a less severe phenotype than *hapln1b* morphants. *Sh3gl3*-MO treated embryos had relatively normal intersomitic vessel development, but displayed reduced circulation as visualized by red blood cell movement in the axial vasculature when compared to controls (data not shown). A closer examination of the axial vessels at 72 hpf revealed a significant reduction in the diameter of the dorsal aorta with a concomitant increase in the diameter of the posterior cardinal vein in morphants (arrow in Figure 6H compared to 6G). This constriction of the dorsal aorta was likely the cause of the observed decrease in circulation. 61 of 83 (73.5%) embryos injected with *sh3gl3*-MO displayed the shrunken dorsal aorta/reduced circulation phenotype while none of the standard control MO injected (5 ng, 0 out of 70 embryos) or uninjected control embryos did. The demonstration that *hapln1b* or *sh3gl3* knockdown results in distinct vascular phenotypes suggest that other genes identified in this gene expression screen will be functionally important and should be studied in further detail.

## Discussion

The transcription factor Etsrp is a critical mediator of vasculogenesis, angiogenesis, and primitive myelopoiesis in zebrafish. In this study we have used gene expression profiling to identify a group of genes that are induced by *etsrp* OE during early development. Among these genes are many previously identified vascular and myeloid genes, supporting the previous identified function of *etsrp* in vascular and myeloid development. The array data was validated by WISH in *etsrp* OE embryos. Gene expression was then examined at 24 hpf for expression in vascular or blood lineages. Genes that met these criteria were then assayed for expression in embryos with *etsrp* knocked down by morpholino antisense. Fourteen genes were found to be both ectopically induced by *etsrp* OE and down regulated in *etsrp* knockdown embryos. Of these genes two, *krml2* and *lrrp33*, are found in myeloid cells and the remaining twelve are vascular. Any expression of these genes outside of hematopoietic or vascular tissue was unaffected by *etsrp* knockdown demonstrating the specificity of *etsrp*'s function. Two newly identified vascular genes, *hapln1b* and *sh3gl3*, were then independently knocked down by morpholino antisense. Each knockdown resulted in distinct vascular phenotypes demonstrating the utility of the screening method used and the importance of the genes identified. Together, these studies have identified genes downstream of *etsrp* that are likely to mediate its observed role in primitive myeloid and early vascular development in zebrafish.

The *ER71/Etv2* mammalian homolog of *etsrp* has been shown to mediate vascular development in mice. Given that *etsrp* has a similar function in zebrafish, it is likely that many of the genes identified in this microarray screen will have mammalian homologs functionally important for angiogenesis and/or vasculogenesis. Of the fourteen genes we focused on, ten have confirmed mammalian homologs. The remaining four cannot yet be determined due to uncertainty in the assembly of the zebrafish genomic sequence. However, it is expected that mammalian homologs for these genes will be found in the future.

Interestingly, *ER71/Etv2* is critical for primitive erythropoiesis in mice, while *etsrp* knockdown or mutation in zebrafish does not affect primitive erythropoiesis. The data presented here supports the previous finding that *etsrp* regulates primitive myelopoiesis and vasculogenesis but not erythropoiesis since genes related to the former group are highly induced by *etsrp* OE while no erythroid genes are induced. So it appears that *ER71/Etv2* and *etsrp* have evolutionarily divergent functions in the context of primitive erythropoiesis. One possible explanation for this difference is that zebrafish primitive erythropoiesis may not progress through an endothelial cell intermediate while mammalian primitive erythropoiesis in the yolk sac blood islands does. Alternatively, the evolutionary function of *etsrp* in primitive erythrocytes may have been co-opted by another ets transcription factor in zebrafish, possibly due to a historical genome duplication event [58]. Another possibility is that the observed difference is due to differential timing of primitive erythrocyte cell death in *etsrp/ER71/Etv2* null fish and mammals. In *etsrp* mutant fish, primitive erythroid cells marked by *gata1* expression are initially specified but then later lost through apoptosis [15]. In *ER71* null mice, no primitive erythrocytes are observed but an increase in apoptosis was noted [18]. This suggests the possibility that primitive erythrocytes are initially specified in *ER71* null mice, but quickly die due to compromised vasculature as happens in fish.

An issue arising from these studies is that *etsrp* OE results in the induction of many nonhematopoietic and nonvascular genes (Figure S2). It is likely that *etsrp* OE directly mis-targets genes normally regulated by other Ets family members. When overexpressed at abnormally high levels it might also overcome repressive mechanisms and thereby transactivate non-specific genes. Included in the list of genes ectopically induced are many *hox* genes that may represent a secondary response to *etsrp* OE. We have found that *krml2* is expressed in myeloid cells and axial vasculature. It was reported that the expression of *hoxb3* was induced by *krml2* overexpression [59]. In this study, not only is *krml2* induced, but *hoxb3* is also induced about five-fold. Ectopic induction of nonvascular/nonmyeloid target genes may also be the result of secondary effects of *etsrp* overexpression. A possible mechanism is that *etsrp* induces premature differentiation of vascular and myeloid cells and these cells signal to adjacent cells to differentiate into additional nonvascular/nonmyeloid cell types. The strong ectopic induction of genes in the early embryo emphasizes that Etsrp is a very potent, early acting transactivator whose roles in gene regulation must be tightly controlled.

It should also be noted that the overexpression of *etsrp* alone is sufficient to induce many other transcription factors such as *scl* and *fli1a*. The overexpression of *scl* has also been demonstrated to induce other blood and vascular specific genes alone [60]. Recently, it was proposed that *fli1a* appears to be at the top of the transcriptional network driving blood and vascular development. Constitutively active *fli1a,* in which the artificial trans-activation domain of the viral VP16 protein was fused to full-length *fli1a* coding sequences, can ectopically induce blood and vascular genes [61]. However, overexpression of *fli1a* alone is not sufficient to induce blood or vascular markers as demonstrated here for *etsrp*. This argues that *etsrp* is more likely the top regulator of hemangioblast development.

The two *etsrp* target genes, *hapln1b* and *sh3gl3*, examined by morpholino gene knockdown each displayed interesting vascular phenotypes. Hapln1b is a member of a protein family that functions as extracellular linkers for hyaluronans and proteoglycans. *Hapln1b* MO treated embryos display truncated intersomitic vessels and disorganized caudal vascular plexus. This phenotype is potentially due to decreased angioblast cell migration or adhesion due to extracellular matrix problems. Future studies will be necessary to establish the exact cellular defect responsible for the vascular phenotype observed in *hapln1b* morphants. *Sh3gl3* is an intracellular protein whose homologs have been suggested to regulate endocytosis and intracellular membrane trafficking [62]. The phenotype observed in the *sh3gl3* morphant, reduced dorsal aorta and increased posterior cardinal vein diameter, is suggestive of disrupted notch signaling as notch signaling mediates artery/vein specification and relative size [63]. Indeed endocytotic mechanisms are critical for proper notch signaling [64]. We hypothesize that *sh3gl3* may regulate artery/vein size and notch signaling through receptor endocytosis.

In summary, we have identified a set of genes induced by *etsrp* OE in zebrafish embryos. Within this set we have identified multiple genes that have restricted expression in the vascular or myeloid lineages and are reduced by morpholino knockdown of *etsrp*. These genes are likely to play important roles in the development of their respective tissues. Additionally, this data can be used in the future to clarify gene hierarchies necessary for hemangioblast differentiation by comparing with mammalian stem cell or zebrafish mutant, morphant, or overexpression gene array data, as we have already done with *cloche*. The results presented here provide a basis for future studies of the hemangioblast.

## Materials and Methods

### Microarray

*flk1:gfp* transgenic embryos were injected with 75 pg of synthetic *etsrp* mRNA, transcribed with Ambion's mMessage mMachine T3 polymerase kit from XbaI linearized *etsrp*-T3TS as previously described [19], and harvested at ∼80% epiboly in pools of 70 embryos per group. The transcription profile of uninjected transgenic embryos was used as controls. Total RNA was isolated with Trizol reagent (Invitrogen), according to manufacturer's instructions. The detailed protocol for microarray hybridization and normalization procedures were described by Horak et al [65] and are available upon request. First-strand cDNA probes were generated by aminoallyl dUTP incorporation and then coupled to either Cy3 or Cy5. The resulting cDNA probes were purified, concentrated, and then hybridized to arrays. Each chip contains 34,647 printed oligo elements (Compugen, Operon, and MWG) designed from zebrafish EST assemblies and representing approximately 20,000 genes, approximately 60% of the total predicted genes according to the public Ensembl database. After hybridization, the slides were washed, dried, and scanned using an Agilent DNA microarray scanner (Agilent Technologies) at 635 nm (Cy5) and then at 532 nm (Cy3). Fluorescence intensities were quantified using Agilent feature extraction software (Agilent Technologies), and changes in expression levels were determined by comparing the signal intensities between *etsrp* OE and uninjected control groups. Hybridizations were performed four times, switching the fluorescent labeling to eliminate biases caused by the labeling process. Samples were normalized using the Lowess calculations and cutoffs for significance were set at a two-fold change in either direction. Oligo sequences were mapped to multiple databases, including RefSeq, UniGene, Ensembl, TIGR, and genomic coordinates to maximally determine gene identity and function. Data were deposited into searchable FilemakerPro and Excel databases for analysis.

### Quantitative RT-PCR

Total RNA was purified with Trizol reagent (Invitrogen) from equal numbers of embryos between experimental groups, and single strand cDNA was synthesized with Superscript II reverse transcriptase (Invitrogen). Real-time PCR was performed using the iCycler iQ Real-Time PCR Detection System (BioRad, Hercules, CA) with iQ SYBR Green Supermix (BioRad). Gene expression levels were measured by the ΔΔ C_t_ method, comparing *etsrp* OE or H2B-mRFP injected groups relative to uninjected controls, with *β-actin* used as the reference gene. Pre-microarray validation was performed from the same samples used for the microarray experiment, and biological triplicates were examined for the injection control experiment reported in Figure S1. Quantitative RT-PCR primers are listed in Table S1.

### Gene cloning and RNA whole mount *in situ* hybridization (WISH)

Genes were cloned into the pCRII Topo vector using the TOPO TA cloning kit (dual promoter) according to manufacturer's instructions (Invitrogen), after amplifying genes of interest from a 24 hpf wild type embryo cDNA library. The primers used are noted in Table S2. Positive clones were analyzed by restriction digest and confirmed by sequencing. WISH was performed as described in [66]. DIG labeled RNA probes were generated by linearizing TOPO-cloned genes with restriction endonuclueases (New England Biolabs), and transcribing with SP6 or T7 RNA polymerase (Promega).

### Embryo injections

Injections were performed with borosilicate glass microcapillaries (Harvard apparatus) that were pulled with a flaming/brown micropipette puller, model P-97 (Sutter Instrument Co) on a PLI-90 microinjector (Harvard apparatus). For microarray validation by WISH, an *etsrp-mcherry* construct was generated by sub-cloning *mcherry* from *mcherry-tol2* (a gift from Hong Zhang) into pSPORT6 between XbaI and XhoI, and *etsrp* was subsequently subcloned into this vector at the 5′end of *mcherry* between BamHI and XbaI sites. To test the requirement of *etsrp* for the expression of the blood/vascular related genes a mixture of *etsrp* morpholinos, MO1 and MO2 (4 ng each) described in [19] was injected into embryos at 1–2 cell stage, and harvested for WISH at 24–28 hpf. Translation blocking morpholinos targeting the first codon of *hapln1b* and *sh3gl3* were synthesized by Gene-Tools, LLC. *Hapln1b* MO sequence: 5′-GAGCAGGAAGGTCATCTTGATTTAT-3′; *sh3gl3* MO sequence: 5′-TTAAACCCGGCCACTGACATCCTTC-3′; Standard control MO sequence: 5′-CCTCTTACCTCAGTTACAATTTATA-3′.

### Image acquisition and processing

*In situ* stained embryos were further processed by a serial dehydration in ethanol, followed by rehydration into 1×PBS. Embryos were imaged either in 1×PBS on agarose coated dishes, or in 2% methylcellulose in depression microscope slides. Images were captured with a color digital CCD camera (Axiocam, Zeiss) mounted on a dissecting microscope (Stemi SV11, Zeiss) with Openlab 4.0.2 software (Improvision, Lexington, MA). Fluorescence images were obtained with the same equipment but on an Axioskop2 plus compound microscope with 10× and 20× objections. In several cases separate images were captured per embryo and areas in focus were compiled with Adobe Photoshop CS version 8.

## Supporting Information

Figure S1 Quantitative RT-PCR of etsrp overexpression and H2B-mRFP injection controls for selected genes. Relative gene expression levels were calculated for etsrp or H2B-mRFP1 expressing as compared to uninjected control embryos at 80 percent epiboly to gastrulation stages for the genes indicated on the x-axis. The induction of endogenous etsrp (3′UTR amplicon from injections encoding etsrp lacking the 3′UTR), fli1a, scl, yrk, and atxr1/tem8 are statistically significant (p<0.05, Student's t-test). Although slightly increased hapln1b and sh3gl3 are not significantly different from controls.
(2.50 MB TIF)

Figure S2 Genes induced by etsrp overexpression but not expressed in blood or vessels. (A) arhgef9; (B) pchdg; (C) novel2; (D) LOC555375 (expression is in ventral somites not vasculature); (E) cbe2; (F) smoc2; (G) LOC550501; (H) similar to testican; (I) hoxb8b; (J) tex2; (K) c1orf192; (L) c20.orf112; (M) znf385; (N) apolipoB; (O) ptgs1; and (P) hoxc3a. Scale bar: 250 um.
(6.74 MB TIF)

Table S1 Quantitative RT-PCR primers
(0.03 MB DOC)

Table S2 Primers used to clone in situ hybridization probes
(0.09 MB DOC)
