# Supplementary figures and images for: Discovery and Characterization of Novel Vascular and Hematopoietic Genes Downstream of Etsrp in Zebrafish
Source: PLoS One. 2009 Mar 24;4(3):e4994. doi: 10.1371/journal.pone.0004994 (PMC2654924; doi:10.1371/journal.pone.0004994)

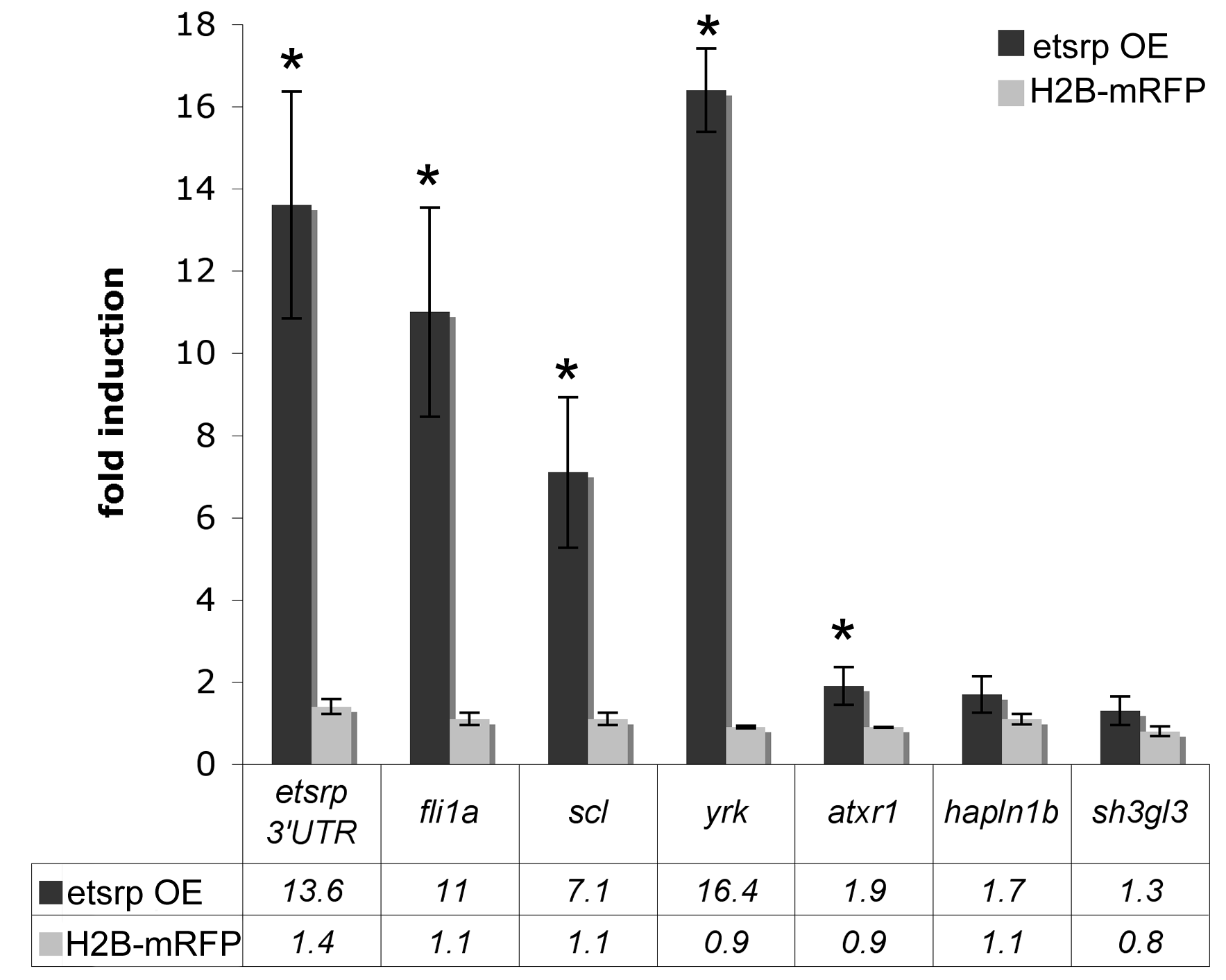

Supplement: Figure S1 — Quantitative RT-PCR of etsrp overexpression and H2B-mRFP injection controls for selected genes. Relative gene expression levels were calculated for etsrp or H2B-mRFP1 expressing as compared to uninjected control embryos at 80 percent epiboly to gastrulation stages for the genes indicated on the x-axis. The induction of endogenous etsrp (3′UTR amplicon from injections encoding etsrp lacking the 3′UTR), fli1a, scl, yrk, and atxr1/tem8 are statistically significant (p<0.05, Student's t-test). Although slightly increased hapln1b and sh3gl3 are not significantly different from controls. (2.50 MB TIF) [file pone.0004994.s001.tif]

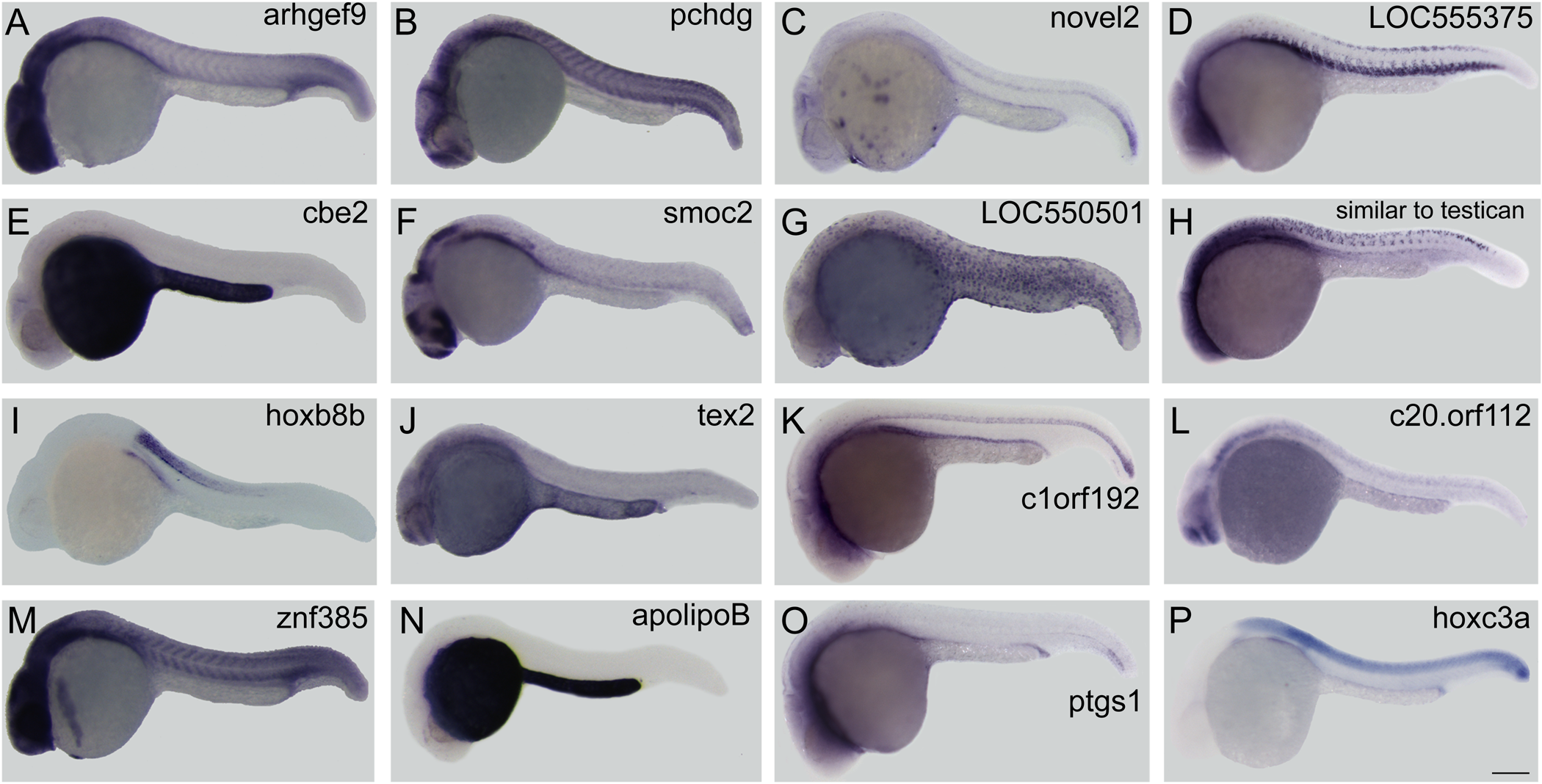

Supplement: Figure S2 — Genes induced by etsrp overexpression but not expressed in blood or vessels. (A) arhgef9; (B) pchdg; (C) novel2; (D) LOC555375 (expression is in ventral somites not vasculature); (E) cbe2; (F) smoc2; (G) LOC550501; (H) similar to testican; (I) hoxb8b; (J) tex2; (K) c1orf192; (L) c20.orf112; (M) znf385; (N) apolipoB; (O) ptgs1; and (P) hoxc3a. Scale bar: 250 um. (6.74 MB TIF) [file pone.0004994.s002.tif]
