# Supplementary material for: Discovery and Characterization of Novel Vascular and Hematopoietic Genes Downstream of Etsrp in Zebrafish
Source: PLoS One. 2009 Mar 24;4(3):e4994. doi: 10.1371/journal.pone.0004994 (PMC2654924; doi:10.1371/journal.pone.0004994)
Supplement: Table S1 — Quantitative RT-PCR primers (0.03 MB DOC) [file pone.0004994.s003.doc]

**Table S1. Quantitative RT-PCR primers**

| Gene | Forward primer | Reverse primer |
| --- | --- | --- |
| flk1 | 5’- CATGTTGGTGGGACACTCAC-3’ | 5’-CTGCAGAGCAGTTGAGGATG-3’ |
| scl | GGAGATGCGGAACAGTATGG | GAAGGCACCGTTCACATTCT |
| β-actin | TGTTTTCCCCTCCATTGTTG | ACATACATGGCAGGGGTGTT |
| etsrp.3UTR | GAGGAATTCTCGAAGGATTGG | TGGTTTTCTAAAGGCACCTAGC |
| fli1A | CCGAGGTCCTGCTCTCACAT | GGGACTGGTCAGCGTGAGAT |
| yrk | CTGAAGCCTTCCTGGATGAG | CTAGCCAGACCGAAATCTGC |
| atxr1/tem8 | GTCGGGGGAATTAAACGAAT | GGTAGTCTGGTGGAGGTGGA |
| hapln1b | GATGGGCTTCCACAAAAAGA | GGTTTGGTGATGGGGTATTG |
| sh3gl3 | GAGCCTTCATCGATCCTCTG | CACTGTGCTCGCAACTGATT |
