# Supplementary material for: Discovery and Characterization of Novel Vascular and Hematopoietic Genes Downstream of Etsrp in Zebrafish
Source: PLoS One. 2009 Mar 24;4(3):e4994. doi: 10.1371/journal.pone.0004994 (PMC2654924; doi:10.1371/journal.pone.0004994)
Supplement: Table S2 — Primers used to clone in situ hybridization probes (0.09 MB DOC) [file pone.0004994.s004.doc]

**Table S2. Primers used to clone selected genes**

| CLONE # PRIMER | PRIMER SEQUENCE | PRODUCT SIZE (Kb) |
| --- | --- | --- |
| Arhgef9.fwd | 5’- GGTCAACGTGATTCTCATATTCTTC -3’ | < 5 |
| Arhgef9.rvs | ACATCCCATGGTTTCTTACATTG |  |
| cbe2.fwd | GGAAAAGCAGCCATGATAAAAAG | 1.7 |
| cbe2.rvs | TTCAGGCTGTGGGAGGTG |  |
| Krml2.fwd | GACGACAAACAGGCTATAGTTTAAGAG | 1.5 |
| Krml2.rvs | TGAAAATGTTACAACTGCTTCGA |  |
| Hoxb8b.fwd | GCGCATCCAATGATACAAGG | 1.1 |
| Hoxb8b.rvs | TTGAATACATGATGCAACAGCC |  |
| Rasgrp3.fwd | TAGAAGTTACACGTCGGATTTGC | 3.3 |
| Rasgrp3.rvs | CCTATCGGCTCAAAGACTCACT |  |
| Lrrp33.fwd | ATGTCAAGCACAAGAGAGACCA | 2.3 |
| Lrrp33.rvs | GAAATGAGATACATTCACATATTTGCA |  |
| Znf385.fwd | **AGCGCGGAGACCAACTTG** | 2.4 |
| Znf385.rvs | TCAGTACGGTGAGAACAAGATTG |  |
| Lrrp51.fwd | GTCTTGACTGTAACTTTTCTATGGTGG | 1.0 |
| Lrrp51.rvs | GTATGTCACGTTACACTGAAAAAAATG |  |
| Smoc2.fwd | AAACTGAATTTCTGACCAGTGTGTT | 1.0 |
| Smoc2.rvs | ATGAAGAACTTTCAACAAAATGATCTG |  |
| Pcdhg.fwd | **TCATTCTCTTGGATTTACAGAATTTTC** | 6.2 |
| Pcdhg.rvs | **TATTAGCATATAAAATGCAAGTAGCAC** |  |
| Novel2.fwd | **AGACTAACACACATTAGCATCCGA** | 0.5 |
| Novel2.rvs | **TAATGACAAAGAATGCCCACAT** |  |
| LOC550501.fwd | TAACAGGACCTTCACTGCTATAAGG | 1.9 |
| LOC550501.rvs | GAAAATTGCAGACATTACAAATGAA |  |
| Tex2.fwd | CACATGTTTCCTTTGGTGTCTAG | 3.3 |
| Tex2.rvs | ACATGTCCTCGAGAGATTCTTTAGG |  |
| apolipoB.fwd | TCTGAAGGCTTCAGCCACTT | 1.5 |
| apolipoB.rvs | CAGAACAAGGATGACAATAGGTTG |  |
| Tem8.fwd | GAGCCGAAGAAGATGTGGAG | 1.4 |
| Tem8.rvs | CTGAGCTTTGGCTGCTTCTT |  |
| C1orf192.fwd | AAGTCGCAAAAGCTCCAGAA | 1.1 |
| C1orf192.rvs | GTGGACCATCACATCCACAA |  |
| Arhgap29.fwd | GTGGCTCTTTGAGCTCCATC | 1.2 |
| Arhgap29.rvs | TCGCACTGAAACACAAGCTC |  |
| Fgd5.fwd | CTGGATCATGTGGGAGTGTG | 1.1 |
| Fgd5.rvs | TGAACAACAGCAGATGATGG |  |
| Ptgs1.fwd | GCTGAAGTGGACGGTGATTT | 1.1 |
| Ptgs1.rvs | AGTGGGTGCCAGTGGTAAAG |  |
| Hapln1b.fwd | CACAGCACAACCCAAAGTGA | 0.9 |
| Hapln1b.rvs | ACGCCGTAGAGTTTGTGCTT |  |
| Loc555375.fwd | GACAATTACCAACCCGGAGA | 0.6 |
| Loc555375.rvs | TGGTCTCCACATGCTCAGTC |  |
| Sim.to testican3 fwd | TGAAGATGCTTGTGCTGGTC | 1.1 |
| Sim.to testican3 rvs | CGTCTCCCATTTCCTCTTCA |  |
| Yrk.fwd | GGAAGGCCAGTAAAGCTGTG | 1.3 |
| Yrk.rvs | ATGCCGAAAGACCATACGTC |  |
| Sh3gl3.fwd | GGCCGGGTTTAAGAAACAGT | 1.0 |
| Sh3gl3.rvs | GAATGATGATGTCGCCCTCT |  |
| Ldb2.fwd | CACGACCCCTTCTACTCGTC | 1.1 |
| Ldb2.rvs | GCTGTTCCAGGGACTGTTGT |  |
| Sim.hemicentin.fwd | TTGGGAAAGCCTCACTTTTG | 1.1 |
| Sim.hemicentin.rvs | TCACATCCGACCAATCAGAA |  |
| Sim.costim.fwd | TTCCTGTGGACTTTGCCAGT | 1.2 |
| Sim.costim.rvs | AGGGCCTTTTCCTGTTGAAT |  |
| EST:AI721944.fwd | TGCAGCCCAATTAAGTGACA | 0.5 |
| EST:AI721944.fwd | GTGCACCTTGAGTTTTGCAC |  |
| EST:AW019729.fwd | TGCAAGTGTTGCCTTTAATGTT | 0.6 |
| EST:AW019729.rvs | CCACAAATATTCCTCATAATCCTTTT |  |
| Hoxc3a.fwd | GCCACCACGTGACTACATTG | 1.4 |
| Hoxc3a.rvs | TGCCTTGGGAAAAGTAGCAT |  |
| C20.orf112.fwd | TTTTTGATATATAGCACACGGTTTT | 0.4 |
| C20.orf112.rvs | AAATATTGGCAACCCATTTCC |  |
